# The Visual Word Form Area compensates for auditory working memory dysfunction in schizophrenia

**DOI:** 10.1038/s41598-020-63962-0

**Published:** 2020-06-01

**Authors:** Alexander B. Herman, Ethan G. Brown, Corby L. Dale, Leighton B. Hinkley, Karuna Subramaniam, John F. Houde, Melissa Fisher, Sophia Vinogradov, Srikantan S. Nagarajan

**Affiliations:** 1 Department of Radiology and Biomedical Imaging, University of California, San Francisco, San Francisco, CA United States; 2 UCB-UCSF Graduate Program in Bioengineering, University of California, Berkeley, Berkeley, CA United States; 3 Department of Psychiatry, University of Minnesota, Minneapolis, MN United States; 4 San Francisco Veterans’ Affairs Medical Center, San Francisco, CA United States; 5 Department of Neurology, University of California, San Francisco, San Francisco, CA United States

**Keywords:** Schizophrenia, Schizophrenia

## Abstract

Auditory working memory impairments feature prominently in schizophrenia. However, the existence of altered and perhaps compensatory neural dynamics, sub-serving auditory working memory, remains largely unexplored. We compared the dynamics of induced high gamma power (iHGP) across cortex in humans during speech-sound working memory in individuals with schizophrenia (SZ) and healthy comparison subjects (HC) using magnetoencephalography (MEG). SZ showed similar task performance to HC while utilizing different brain regions. During encoding of speech sounds, SZ lacked the correlation of iHGP with task performance in posterior superior temporal gyrus (STGp) that was observed in healthy subjects. Instead, SZ recruited the visual word form area (VWFA) during both stimulus encoding and response preparation. Importantly, VWFA activity during encoding correlated with the magnitude of SZ hallucinations, task performance and an independent measure of verbal working memory. These findings suggest that VWFA plasticity is harnessed to compensate for STGp dysfunction in schizophrenia patients with hallucinations.

## Introduction

Schizophrenia is a debilitating psychiatric illness characterized by a range of neurocognitive deficits and corresponding abnormal neural circuit activations^1–4^. Patients exhibit auditory processing and verbal memory deficits associated with widespread disturbances in frontoparietal and frontotemporal circuits, and these impairments show a significant relationship with functional outcome^2,5–8^. Characteristic findings include abnormalities in lower-level neurophysiological operations such as auditory mismatch negativity^9–12^, temporal integration of speech sounds^13^, and tones and prosody discrimination^14,15^. Abnormalities are also seen across fronto-temporal networks subserving auditory, verbal working memory, encoding, and retrieval functions^16–19^.

In particular, patients who suffer from auditory hallucinations exhibit worse verbal working memory deficits than non-hallucinators even after controlling for auditory attention and perception^20^. During the first acute psychotic episode of schizophrenia, verbal working memory deficits predict levels of auditory hallucinations^21^. Additionally, auditory hallucinations are associated with structural and functional abnormalities in temporal cortex/planum temporale^6,22–28^.

Despite these widely reported impairments in speech-sound representation that appear fundamental to the disease process, many patients with schizophrenia spectrum disorders perform at or above the level of matched healthy controls on auditory and language tasks, raising the possibility that compensatory pathways may have developed^29^. People at risk for psychosis who display performance comparable to controls exhibit evidence of both impaired and compensatory neural processes^30^. Extensive neural system compensation and re-organization in response to acquired brain lesions are known to occur in other conditions, particularly when the lesions occur early in development^31–34^. However, cross-modality appropriation of cortical resources in response to neurodevelopmental dysfunction has been little studied in schizophrenia or any other psychiatric illness.

In the present study, we exploit the high spatiotemporal resolution of magnetoencephalography (MEG). We have previously shown that when healthy subjects engage in an auditory speech reproduction task, induced high gamma power (iHGP) fluctuates across the dorsal speech stream in a spatially and temporally choreographed manner that correlates with performance^35^. High gamma amplitude reflects local neuronal activity action and dendritic potentials^36^, encodes task variables similarly to single and multiunit activity^37^, and captures language and cognitive processes in human MEG experiments^38,39^. High gamma oscillations are of particular interest in this patient population because gamma band abnormalities appear to be related to auditory processing fidelity^29^, working memory deficits^40,41^, and symptom severity^42–45^. In the present work, we test the hypothesis that patients with schizophrenia recruit ancillary neural ensembles to support auditory working memory, reflected in iHGP signal dynamics. We took advantage of the symptom scales and neuropsychological battery administered to these patients to further identify activations that differ with disease state. Specifically, we examined whether activations that differed at a group level between patients and controls also varied as a function of hallucination level. We hypothesized that in addition to dysfunctional activation patterns we would find evidence of facilitatory processes that may reflect compensation for underlying disease-related pathology in audio-motor speech areas.

## Results

### Behavior

Subjects performed an auditory syllable reproduction task as previously described in healthy controls^35^. Subjects’ accuracy and reaction time did not differ between control and patient groups (Linear Mixed Effects Model, omnibus test for between group differences, Wilks *F(1,51)* = 0.7693; accuracy *F(1,51)* = 1.89, *p* = 0.18; reaction time *F(1,51)* = 0.086, *p* = 0.77) but varied with task difficulty within-group. Both patients with schizophrenia (SZ) and healthy controls (HC) performed significantly better on the two-syllable than on the four syllable trials (HC: 92 +/− 2% vs. 66 +/− 5%; Tukey-Kramer post-hoc tests *p* < 10^−8^; SZ: 88 +/− 2% vs. :51 +/− 4%; Tukey-Kramer post-hoc tests *p* < 10^−7^). Reaction times were different between two-syllable reproduction (HC: 0.64 +/− 0.04 s, SZ: 0.63 +/− 0.03 s) and four-syllable reproduction (HC: 0.83 +/− 0.06 s, SZ: 0.85 +/− 0.03 s) (Tukey-Kramer post-hoc tests *p* < 7 × 10^−4^, 2 × 10^−6^ respectively). Additionally, a MANOVA, with a hallucination score as a dependent variable, did not show any significant differences in either reaction time or accuracy.

#### Patients with schizophrenia show reduced stimulus-induced iHGP in left dorsolateral prefrontal cortex (DLPFC) but increased stimulus-induced HGP in left fusiform gyrus (Visual Word Form Area, VWFA)

**Figure 1:**
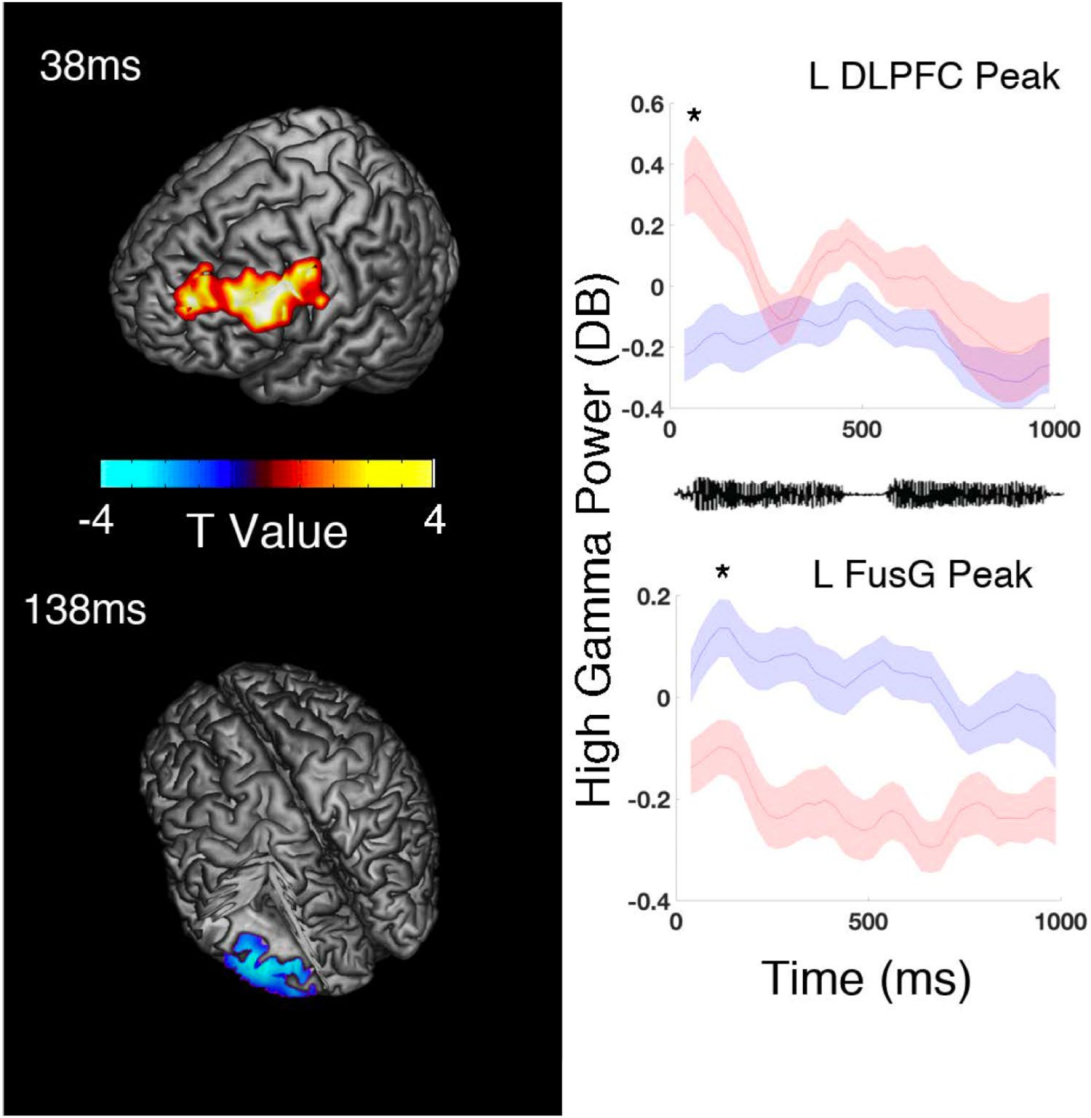
Induced High Gamma Power (iHGP) differences during syllable encoding between healthy controls (HCs) and individuals with schizophrenia (SZ). HCs activated DLPFC during stimulus encoding while SZ did not. The peak difference occurred immediately after stimulus onset (37.5 ms T = 4.9 *p* < 0.0004 Bonferroni *p* < 0.05). Next, SZ activated the Visual Word Form Area (VWFA) in the left fusiform gyrus during stimulus encoding while HC did not. The peak differences occurred immediately after the first syllable (137.5 ms T = −4.6 *p* = 0.0005 Bonferroni *p* < 0.05) and second syllable (637.5 ms T = −4.4, *p* = 0.0002 Bonferron*i p* < 0.05). Asterisks indicate the times of peak statistical significance and accompanying neural renderings. Positive T-values (warm colors) in neural renderings represent greater power in the HC group, while negative T-values (cold colors) reflect greater power in the SZ group.

**Figure 2:**
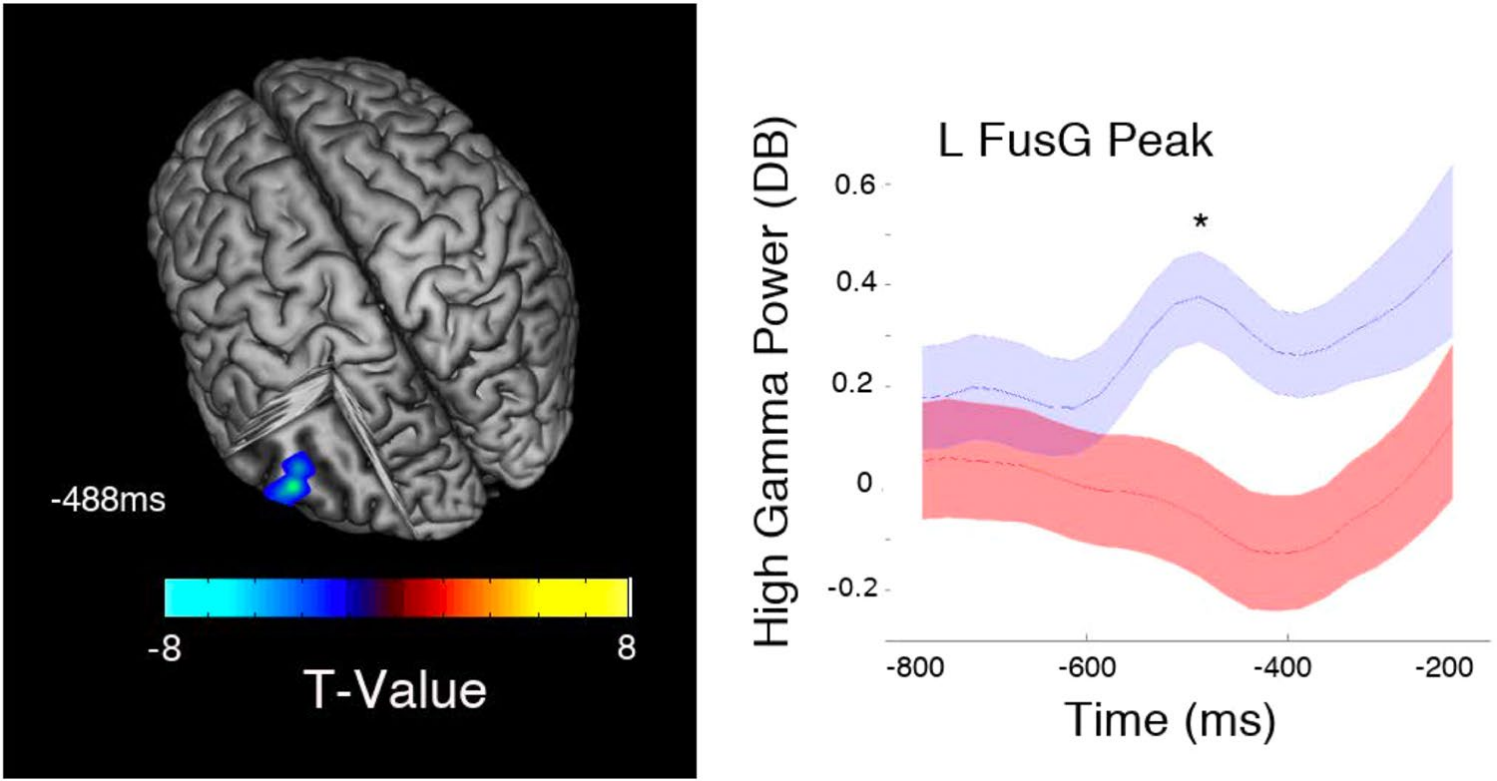
HC-SZ HPG during the pre-response period, VWFA iHGP was greater in SZ than in HC during the pre-response recall period; peaking at 487.5 ms before response onset (T = 7.8 *p* = 0.0002 FDR *p* < 0.05). Asterisks indicate the time of peak statistical significance between the two groups and the accompanying neural renderings.

The comparable behavioral performance of patients and controls allowed us to look for neural mechanisms operating in the brains of schizophrenia patients but did not allow for controls that might reflect compensation. We compared beamforming source-localized iHGP changes (100 ms sliding windows with 25 ms overlap) across the entire cortex in the two groups during speech syllable encoding (Fig. 1). In our initial whole-brain, all voxel analysis, we found differences in two regions: the left DLPFC and the left fusiform gyrus (FusG) that survived Bonferroni correction for multiple comparisons. HCs exhibited greater stimulus-induced iHGP in DLPFC immediately after first syllable onset, peaking at 37.5 ms and persisting until 212.5 ms (MNI [−55 25 30], *T* = 4.9, *p* = 0.0004 Bonferroni < 0.05). The schizophrenia sample subjects showed an iHGP response in left FusG during each syllable that was absent in controls, peaking relative to controls at 187.5 ms (MNI [−45 −55 −20], *T* = −4.6, *p* = 0.0005 Bonferroni < 0.05) and again at 637.5 ms (MNI [−45 −50 −15], *T* = −4.4, *p* = 0.0002 Bonferroni < 0.05). The first FusG peak was localized within 1 functional voxel and the second within 2 voxels of the canonical center of the VWFA (MNI Coordinate of [−45 −55 −15])^34^. Independently, when we time locked to vocal-response and performed a whole-brain analysis, significant differences emerged only in a similar left FusG area (also within 5 mm of VWFA) peaking 487.5 ms before voice onset (MNI [−50 −60 −15] *T* = −7.8, *p* = . 0002 FDR < 0.05, Fig. 2). For further analyses we selected the time windows with peak iHGP within these statistically significant time ranges.

#### Schizophrenia patients with higher hallucination scores show greater VWFA activation during stimulus encoding

We hypothesized that abnormal patterns of VWFA activity during syllable reproduction in SZ may be associated with compensation for disease related dysfunctions. To test this hypothesis, we examined whether VWFA activation was associated with hallucination scores on the PANSS battery using a generalized linear model (GLM). We then tested whether task performance was predicted by VWFA activation and/or hallucination score within the SZ group using analysis of covariance (ANCOVA).

**Figure 3:**
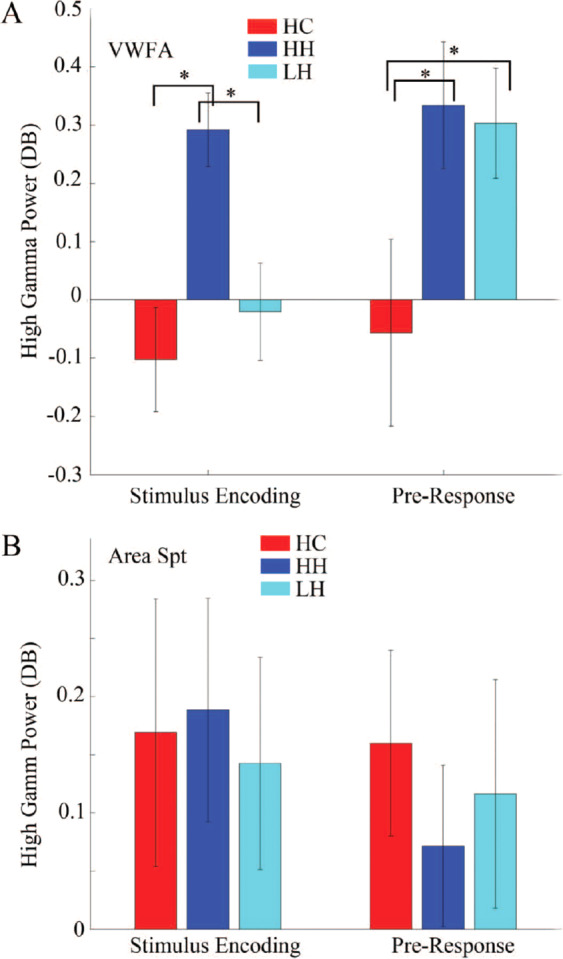
Activations in VWFA vary with burden of hallucinations. (**A**) VWFA activations: During stimulus encoding only the high hallucination (HH) subgroup exhibited a significant activation of the VWFA. During the pre-response period both the HH and the low hallucination (LH) group activated the VWFA. Further, VWFA iHGP in the HH group, was significantly greater than both the LH and the HC (one-way ANOVA, F = 7.49, *p* = 0.0019). (**B**) Activations in posterior superior temporal gyrus (STGp) did not differ significantly between the HC, HH, and LH groups in either the stimulus encoding or the pre-response period.

During encoding of the first and second syllable stimuli, peak VWFA iHGP during the first (β = 0.75 *p* = 0.018) and second syllables (β = 0.48 *p* = 0.019) correlated positively with hallucination scores even after controlling for accuracy. Furthermore, VWFA activity and hallucination score predicted task performance. Peak iHGP predicted accuracy at a trend level during the first syllable (Slope = 2.14, *A*_*ccuracy*_ = 2, *F*_*Accuracy*_*(1)* = 4, *p* = 0.054, *F*_*Hallucination*_*(5)* = 1.72, *p* = 0.16,) and both iHGP and hallucination score predicted accuracy significantly during the second syllable (Slope_*Accuracy*_ = 4.6 *T* = 3.38, *F*_*Accuracy*_*(1) = 11.4, p* = *0.0021, F*_*Hallucination*_*(5)* = 2.54, *p* = 0.047). To aid visualization and interpretation, we divided the patients into those with more severe hallucinations (“high hallucinations”) and those with low hallucination scores (“low hallucinations”). VWFA activation was significantly greater in the high hallucination group than in the low hallucination or control group (one-way ANOVA, *F* = 7.49, *p* = 0.0019; Fig. 3). Furthermore, only patients with high levels of hallucinations showed a clear activation peak, which was absent in the low hallucination group. Interestingly, DLPFC activation did not differ significantly between high and low hallucinations, even at an uncorrected threshold of *p* < 0.05 at any time point. To assess laterality in the ventral visual stream activation in the high hallucination group, consistent with engagement of the left lateralized VWFA, we compared the peak activation in the left fusiform gyrus with the peak activation in the right fusiform gyrus. The left fusiform gyral activation was significantly greater than the right (*T* = 2.1, *p* = 0.0457), confirming that left lateralization was not significant in the broader SZ group or the HC. The pre-response VWFA peak was also greater in the high hallucination group than in the low hallucination group (*T* = 3.1, *p* = 0.003).

#### In schizophrenia patients with high hallucinations, VWFA activity, but not posterior Superior Temporal Gyrus (STGp) activity, is correlated with auditory working memory task performance

We previously found that in healthy individuals, iHGP levels in the speech auditory-motor network correlated with behavioral performance during syllable encoding and response preparation during this auditory working memory task^35^. In particular, activity in the left STGp correlated with accuracy during both the stimulus (peaking at 512.5 ms) and pre-response periods (peaking at −712.5 ms and −287.5 ms). We queried whether iHGP levels in STGp would show the same relationship to behavior in SZ participants and whether activity in the VWFA might be related to task performance in SZ. We defined the VWFA for each group as the peak voxel within 5 mm (one functional voxel) of the canonical VWFA, consistent with recent work^46^.

**Figure 4:**
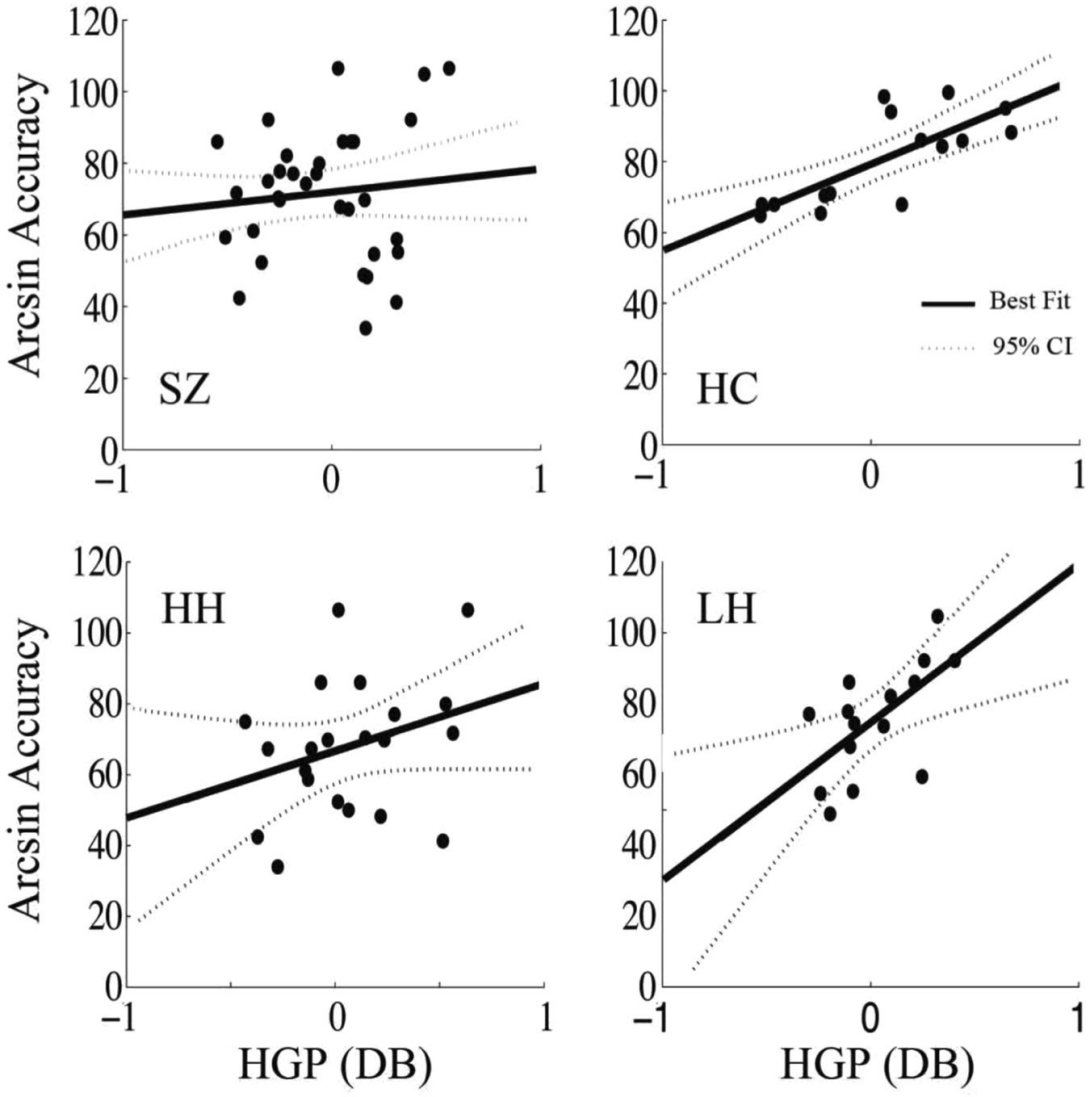
iHGP-auditory working memory task performance correlations in STGp. In the entire SZ sample there was no correlation between induced iHGP in STGp and accuracy. LH showed a similar relationship between iHGP and performance in STGp as HC: (LH: peak time 712.5 ms, r = 0.77 *p* = 0.003; HC: peak time 512.5 ms, r = 0.75 *p* = 0.0003). However, HH showed no significant correlation between iHGP and performance in STGp.

In the full schizophrenia sample, iHGP in STGp showed no significant correlation with performance, and the slope differed significantly from the controls (Fisher r to z, *z* = 2.2, *p* = 0.03). On the other hand, VWFA iHGP did show a significant correlation with performance at 662.5 ms during stimulus encoding (*r* = 0.47, *p* = 0.004, Fig. 4), and a trend toward significance at 137.5 ms (*r* = 0.31, *p* = 0.07). When we divided the full schizophrenia sample by level of hallucinations as above, the low hallucination group demonstrated iHGP in left STGp that showed a significant relationship to task accuracy (*r* = 0.68, *p* = 0.01, Fig. 4), similar to what we observed in the HC (Fisher r to z, *z* = 0.94, *p* = 0.35). However, the high hallucination group showed no correlation between iHGP in STGp and task performance, and the slopes differed significantly (Fisher r to z, *z* = 2, *p* = 0.04).

**Figure 5:**
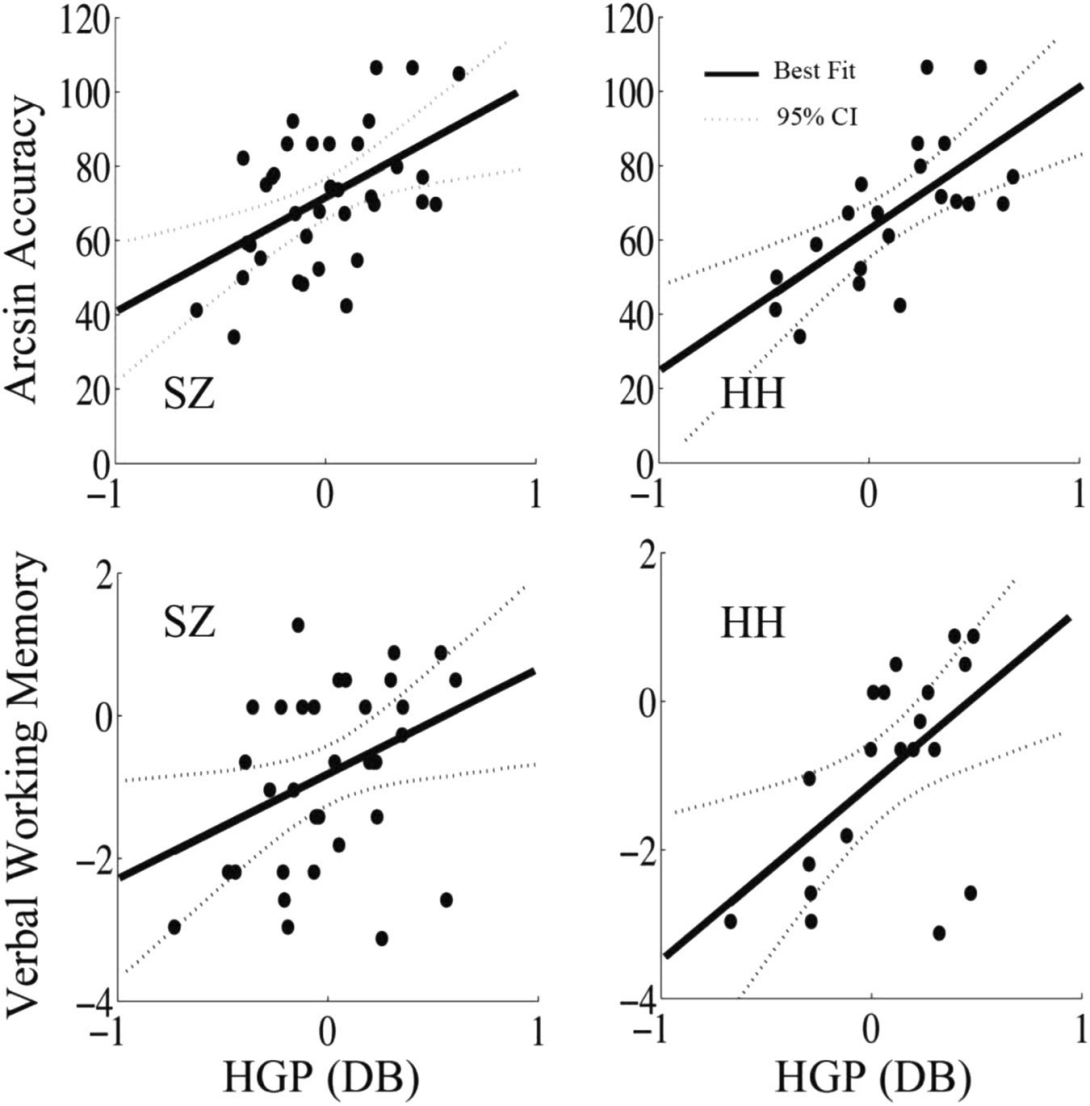
iHGP-behavioral correlations in the visual word form area (VWFA). A) Both the SZ and the HH groups showed positive correlation between iHGP and task performance (SZ peak at 662.5 ms, r = 0.47, *p* = 0.004; HH peaks at 137.5 ms r = 0.67 *p* = 0.001 and 637.5 ms r = 0.46 *p* = 0.04). B) Both the SZ and HH groups showed positive correlation between iHGP and verbal working memory (SZ peak at 212.5 ms, r = 0.45 *p* = 0.006; HH peak at 237.5 ms, r = 0.57, *p* = 0.009).

In contrast, when we examined the VWFA, we found a strong relationship between iHGP and task performance in both the full schizophrenia group and the high hallucination group during both the first syllable (*r* = 0.67 *p* = 0.001) and second syllable encoding periods (*r* = 0.46, *p* = 0.04) (Fig. 5). Considering the dissociation between STGp and accuracy in these subjects, engagement of VWFA may represent a compensatory response to dysfunction in STGp. While neither healthy subjects nor low hallucinators activated the VWFA on average during stimulus encoding, iHGP in the VWFA correlated with performance in both these groups, suggesting that high-performing subjects may engage VWFA to help boost performance, but that VWFA activation during auditory working memory processing in these groups does not represent the same compensatory process as in the high hallucinators. During the response preparatory period, VWFA iHGP did not correlate significantly with task performance in any of the subject groups.

#### In the high hallucination, but not low hallucination group, VWFA activity correlates with performance on a standard neuropsychological test of verbal working memory

In order to further assess whether VWFA activation in schizophrenia subjects represents a true compensatory adaptation, we regressed scores from a standard neuropsychological test of verbal working memory (VWM, the Letter-Number Span; see Experimental Procedures) against iHGP. Across all patients, ANCOVA analysis showed a correlation between the second syllable peak iHGP and VWM (Slope = 0.54, *T* = 2.12, *F* = 14.65, *p* = 0.042). Dividing into two groups by hallucination score as before, in the high hallucination group VWFA iHGP correlated with VWM scores during the first (*r* = 0.46, *p* = 0.06) and second (*r* = 0.62, *p* = 0.02) syllable encoding periods (Fig. 5). STGp responses did not show a correlation with performance. In contrast, in healthy controls, a highly significant correlation between iHGP and VWM scores occurred in the left STGp during the second syllable encoding, consistent with the correlation observed in controls with auditory working memory task performance (*r* = 0.82, *p* = 0.0002).

## Discussion

We present evidence here of compensatory activation of the Visual Word Form Area (VWFA) in the left ventral visual stream during auditory working memory in patients with schizophrenia. Performing a whole-brain analysis in cortical source space, we found that patients with schizophrenia exhibited activation of a small region of the left fusiform gyrus during each syllable of phoneme stimulus encoding, as well as preceding verbal repetition. Such responses were not seen in healthy controls. Narrowing to this region of interest, which overlaps tightly with the canonical VWFA, we found that the response correlated with the level of participant reported hallucinations. The activation level and hallucination score independently predicted performance. VWFA response also correlated with an independently assessed standard neuropsychological measure of verbal working memory. Furthermore, patients with high levels of hallucinations lacked the normal correlations between auditory working memory task performance and activation in STGp.

Taken together, our findings suggest that ventral visual cortex, and specifically the VWFA, has been co-opted in patients with disabling auditory hallucinations to support a cognitive function normally reliant on the dorsal speech stream. As in a recently published syllable detection task in the same cohort^47^, our patients achieved similar behavioral performance as the healthy controls, likely due to the moderate level of difficulty, allowing us to look for direct evidence of compensatory processes. A similar approach has been recently used to find compensatory activation during working memory in subjects at high risk for psychosis^30^. While that study found increased prefrontal engagement, our results represent a novel finding of adaptive cross-sensory plasticity.

In healthy subjects, activation of areas lying along the dorsal speech stream underlies the verbal reproduction of heard speech sequences. The activation of those areas occurs in an order that corresponds to the functional stages of the task, and the level of activation modulates with sequence length^35,48,49^. Furthermore, the success of syllable sequence reproduction varies with the level of iHGP in these phonological loop regions in a temporally choreographed manner^35^. For instance, during listening, superior temporal and inferior parietal areas, along with dorsal premotor cortex, mediate the encoding of the target following the stimulus and leading up to the vocal response. An interaction between Broca’s Area and the STGp underlies formation of correct motor plans for production of the sequence.

We found that patients with schizophrenia activated the VWFA during syllable repetition, and achieved a comparable level of task performance as healthy comparison subjects relying solely on the dorsal speech stream. These results are consistent with a prior study that patients with schizophrenia who perform comparably with controls on a working memory display altered brain activation^30^. The peak activation points we found lie within 5–10 mm (1–2 functional voxels) of the classic location of the VWFA in the left fusiform gyrus, the presence of which has been replicated in patients with schizophrenia^50^ and notably posterior and inferior to the multimodal linguistic region in area BA37^34,50–52^.

The VWFA, lying along the ventral visual stream, serves roles in reading, analogous to those played by posterior temporal/inferior parietal areas in listening. As the name suggests, the VWFA is activated in tasks involving the visual perception and remembering of written words. The level of activity varies with the verbal working memory load. The VWFA exhibits a high degree of anatomical connectivity to peri-sylvan areas, supporting its role in language processing^53^. Dysfunction of the VWFA is associated with alexia and dyslexia, much as lesions in the STGp are associated with receptive and conduction aphasia^54–56^. In light of our findings of co-option of VWFA, it is interesting to note that reading impairment is a common finding in schizophrenia and reaches the level of dyslexia^57^; indeed, this is a frequent observation of young patients early in the course of illness when reading becomes increasingly difficult for them. Patients with schizophrenia also exhibit impaired performance with reading visually degraded words^50^, which further suggests a bandwidth ceiling in ventral visual stream utilization, resulting in a trade-off between auditory and visual processing.

Work in congenitally blind adults suggests that in addition to representing visual word input, VWFA may perform a task-specific and sensory modality independent computation, linking letter shapes to phonology^34^. In the Striem-Amit study, blind individuals were trained to “read with sounds” by associating acoustic signals (“soundscapes”) with orthographic features; plasticity in the VWFA was found to underlie this auditory-based learning. Indeed, Dehaene and Cohen^58^ have suggested that reading acquisition partially recycles a cortical territory evolved for object and face recognition; presumably a territory with a high degree of inherent developmental plasticity. Our data suggest that patients with schizophrenia co-opt this region in support of auditory processing, perhaps when STGp fails to function optimally.

Dysfunction in the STGp and its interactions with other brain regions has been implicated as a possible source for verbal working memory deficits and auditory hallucinations in schizophrenia^6,27,59^. In the present work, activation levels in STGp did not differ between high and low hallucination group and healthy comparison subjects. However, neural behavioral correlations in STGp differed significantly between healthy subjects and the whole schizophrenia sample, as well as between healthy subjects and the high-hallucination sub-group. In low hallucination subjects, the peak STGp iHGP during perceptual encoding correlated with performance as it does in normal subjects. While in the high hallucination group within the same time-window, we found no significant correlation between induced iHGP in STGp and performance, although these between-group correlation differences did not reach significance. This similar level of overall activation accompanied by absent/inverted neural-behavioral correlations in the group with high hallucinations suggests dysregulated and possibly maladaptive processing of speech sounds in STGp.

We propose that dysfunction in the neural computations of the STGp likely contributes to the genesis of auditory hallucinations and auditory working memory impairments, as well as drives the compensatory plasticity in ventral visual cortical regions observed here. In this model, VWFA co-option would develop over the early phases of schizophrenia in response to dysfunctions in auditory processing regions that are known to pre-date the onset of the illness^60^. Such a model would be consistent with longitudinal studies that show progressive and interrelated decrease in mismatch negativity^61^, and loss of gray matter volume in STG in first-episode patients associated with progression of psychotic symptoms^62^.

Our findings, showing decreased dorsolateral prefrontal cortex (DLPFC) activity in patients with schizophrenia, are consistent with a large literature showing altered DLPFC function in schizophrenia^63^. Interestingly, these results are not consistent with findings that DLPFC activity is greater in patients with schizophrenia with preserved working memory^30^, however the timing of the difference observed here makes it difficult to compare directly to fMRI studies. While we did not find differences in DLPFC activity between the low and high hallucination groups, possibly due to a lack of power, these results raise the question of whether dysfunction of the dorsal speech stream in schizophrenia may in turn be related to impaired prefrontal top-down functioning more broadly.

While we do not at this point fully understand the clinical consequences of an alternate visual stream pathway for carrying out auditory working memory tasks in high hallucinators, it may have profound implications for the design of effective cognitive training approaches and other neural system-based treatment strategies. The use of auditory vs. visual/lexical modes of training is likely to drive significantly different patterns of cortical plasticity in patients who have intact STGp functioning vs. those who are reliant on “alternate” or “compensatory” networks including the ventral visual stream. Such differential patterns of plasticity may drive quite different behavioral outcomes. For example, we have found in two prior studies of targeted cognitive training of auditory processes that schizophrenia subjects exposed to the visually intensive computer games control condition showed a worsening in their verbal/learning memory performance (see^64–66^).

The present study has notable limitations. The larger clinical study of which this was a part was not designed to look specifically at levels of hallucinations, and thus our examination of the VWFA compensation in this group was necessarily post-hoc, despite being hypothesis driven. Similarly, the sample sizes for the different hallucination-levels are small, even at the aggregate level of low (n = 12) and high (n = 14) hallucination level. In the present cross-sectional sample of patients with medicated chronic disease, medication effects cannot be ruled out and a neurodevelopmental picture cannot be ruled in. Another possible limitation relates to the interpretation of high gamma activity in patients with schizophrenia. Many studies show attenuation of high gamma responses in schizophrenia, although some show regional increases, these changes are thought to relate to underlying disruption in the excitatory/inhibitory balance maintained by pyramidal-inhibitory cell microcircuits that may change over the course of illness^67^. Our work is consistent with this otherwise difficult to reconcile literature, implying that high gamma responses are disrupted in brain areas specifically related to the impairments in schizophrenia, auditory, and prefrontal cortex, while remaining relatively intact in other areas (fusiform gyrus) accessible for compensatory recruitment and increased activation.

Detailed functional connectivity analyses across the illness course will be needed to fully understand the developmental pathway of abnormalities in baseline high frequency neural oscillations, and in task-related interactions between prefrontal and sensory cortices; including the time-course and functional implications of compensatory plasticity in ventral visual cortical pathways. Such studies will help us to address the critical question of whether cognitive training and possibly brain-stimulation methods should focus on restoring function in impaired neural systems and should minimize the use of compensatory pathways, or whether instead the harnessing of compensatory networks should be facilitated. It is almost certain that the treatment implications will be quite different for different subgroups of patients depending on their underlying baseline neural system pathology. A thorough understanding of how sub-network dysfunction, adaptation, and compensatory plasticity occurs across individuals with schizophrenia is the only means by which we will be able to develop and target interventions that are precisely tailored to the underlying neural signature of each patient.

## Materials and Methods

### Participants

As part of a large randomized controlled trial of neuroplasticity-based cognitive training in SZ groups registered at ClinicalTrials.gov NCT00312962, clinically stable and persistently ill volunteer schizophrenia subjects were recruited from community mental health clinics. Healthy comparison subjects were recruited from the larger San Francisco community via posted advertisements, and were matched to patients at a group level on age, education, and gender. All patient participants had outpatient status for 3 months prior to study entry and no significant medication changes (dosage change >10%) during the study. Inclusion criteria were: Axis I diagnosis of schizophrenia (determined by the DSM-IV SCID) or, for healthy subjects, no Axis I or II psychiatric disorder (SCID-NP); no substance dependence or current substance abuse; good general physical health; age between 18 and 60 years; English as first language; free of psychiatric medications. Additionally, no participant showed hearing loss greater than 20 dB via pure tone audiometry at 1000, 4000, 8000 Hz prior to the first session of MEG recording. This study was approved by the UCSF Human Research Protection Program and performed in accordance with the ethical standards specified in the 1964 Declaration of Helsinki.

This report describes a subset of MEG findings obtained from trial participants who were found to be physically compatible with neuromagnetic recording and willing to undergo MEG, and at least one session of MRI recording. After having study procedures explained, participants gave written informed consent and underwent baseline neuropsychological assessments over a 2–3 week period, followed by a battery of auditory tasks during MEG recording and an MRI session prior to beginning the study trial. Findings from other MEG experiments with these subjects have been reported previously^1,29,47^.

### Cognitive, clinical, and functional assessments

Cognition was assessed in SZ and a subset of HC (n = 11) with measures recommended by the Measurement and Treatment Research to Improve Cognition in Schizophrenia (MATRICS) (Kern *et al*., 2008; Nuechterlein *et al*., 2008). Measures were obtained from test publishers, and raw scores were converted to z-scores using normative data stratified by age, and published by the test authors. Symptom severity was assessed in patients with the Positive and Negative Syndrome Scale (PANSS^68^), and functional status with the Quality of Life Scale (QLS): a semi-structured interview with operationalized criteria that permits a reliable and valid assessment of real-world psychosocial and functional performance in schizophrenia^69^.

### Auditory working memory task during MEG recording

Participants were presented with two or four syllables involving mixtures of /ba/, /pa/, and /da/ pre-recorded from a female speaker, each lasting 470 ms with 50 ms inter-stimulus intervals (ISI) (for details see Fig. 1 from)^35^. We asked the participants to listen to the syllables, remember them, and repeat them out loud after a visual cue, which appeared at a jittered delay uniformly distributed between 2050 and 2150 ms post-stimulus onset. The task consisted of 160 trials; 80 four-syllable trials randomly interleaved with 80 two-syllable trials. Each trial lasted approximately six to seven seconds depending on subject reaction time and included a 1 s inter-trial interval. Two staff members separately listened to participant responses and scored correct trials.

### Data acquisition

We recorded neural activity using MEG with a 275-channel sensory array in a magnetically shielded room using the Omega 2000 Whole-cortex system (CTF Systems Inc./VSM Medtech, Ltd. Port Coquitlam, BC, Canada). To correct for distant magnetic field disturbance, we used twenty-nine reference sensors to calculate a synthetic third-order gradiometer^70,71^. We acquired MEG signal at a sampling rate of 1200 Hz and a bandpass filter of 0.001–300 Hz. Radio-emitting coils (fiducial landmarks) were placed on the participant’s nasion, and left and right ear (1 cm anterior to the preauricular point in the plane of the nasion) so head position could be monitored in relation to the sensor array throughout the recording with inclusion dependent on <7 mm translations in head movement. There were no statistically significant differences in head movement between groups.

### Data preprocessing

The data were split into epochs of −1s to 7 s relative to onset of the first syllable. Markers for stimulus and response onset were added and specified as to the type of trial (two- or four-syllable). We rejected channels and trials based on high-frequency (>50 Hz) artifacts that occurred during the time period of interest for each particular analysis. Specifically, trials with high-frequency activity consistently greater than 1.5pT, or in which the participant spoke during this interval were discarded. All incorrect trials were discarded to ensure task participation during all analyzed trials. These quality controls resulted in a minimum of 60 trials per subject. The number of channels and trials removed varied from subject to subject but did not differ significantly between groups (*p* > 0.05).

### Source reconstruction

After preprocessing the data, we localized sources of high gamma (50–120 Hz) neural activity using a time-frequency optimized adaptive spatial filters implemented in the Neurodynamic Utility Toolbox for MEG (NUTMEG) (http://bil.ucsf.edu)^72^. Source reconstruction procedures described here are identical to those reported in our previous publications^35^. To enable neural-source localization, high-resolution anatomical MRIs were obtained for each subject and spatially normalized to a standard MNI template brain (http://www.fil.ion.ucl.ac.uk/spm/software/). Forward models of potential dipolar source locations (voxels) were generated with individual MRIs (in subjects’ native space). Whole-head source reconstructions were then computed with 5 mm voxel grids and 100 ms sliding windows (fixed for the control periods) with a 25 ms overlap using adaptive spatial filtering methods. In order to avoid mis-localizations due to strongly and temporally correlated sources between the two hemispheres, data from sensors above each hemisphere were analyzed separately 74^73^. For activations, noise-corrected pseudo-F ratios were computed between active windows (i.e. intervals before subjects’ verbal response or intervals after stimulus onset) and a pre-stimulus baseline control window. For contrasts between the four and two syllable conditions, noise-corrected pseudo-F ratios were computed for sliding windows covering the time-periods of interest for both conditions. For the inter-trial interval baseline analysis, we averaged the iHGP in each 100 ms window across the 1 s inter-trial interval for each voxel for each individual subject, and then averaged across subjects as below. Localizations were computed using the shared computing cluster at the California Institute for Quantitative Biomedical Research (www.qb3.org). Source reconstructions were subsequently normalized to the MNI template for group analyses.

### Statistics for group-level activations and neurobehavioral correlations

We included the 36 SZ subjects and 17 HCs subjects that passed data pre-processing checks detailed above and in prior publications in group contrasts using standard nonparametric randomization tests^35,72^. The SZ subjects consisted of two randomized sub-groups separately matched to the HCs for the purposes of a cognitive training experiment, but combined here to increase power. The distribution of source reconstructed brain activity for each subject was spatially normalized to the standard MNI template brain. The spatial normalization enabled group analyses to be performed in a common spatial grid using statistical nonparametric mapping. Briefly, the three-dimensional average and variance maps across subjects were calculated at each time-frequency point and smoothed with a 20 × 20 × 20 mm^3^ Gaussian kernel. From this image, a pseudo-t statistic was obtained at each voxel and time window for each frequency band. SnPM based null distributions were created by permuting voxel labels (2 ^N^ permutations with N being the number of subjects). The resulting *p*-values across time, frequency, and voxels were corrected for multiple comparisons as described using the Bonferroni Procedure or the False Discovery Rate (FDR) procedure with a threshold of 5%^74,75^.

We performed two types of analyses: (1) computation and comparisons of task-induced high gamma power time locked to either the stimulus or the response at a whole-brain and ROI level, and (2) regression of induced high-gamma power in active task with behavioral metrics and hallucination score. To reveal post-stimulus locked differences in activation at the whole-brain level, we included all 36 SZ and 17 HC subjects that passed basic quality controls. We performed ANCOVA ROI analyses on this sample with high gamma power and task accuracy as continuous variables and PANSS hallucination score as an ordinal covariate. After applying the same data quality and trial number thresholding as for all SZ and HC (see below), the result was 15 subjects with hallucination scores between 1–3 (“not present”, to “mild”, termed “low hallucinators/LH”) and 21 between 4–6 (“moderate” to “severe”, termed “high hallucinators/HH. To control for differences in overall task performance as a function of hallucation score for iHGP comparisons, we set a minimum accuracy limit of 60% or higher. The result was the largest sample with statistically indistinguishable task performance between groups and included 12 LH, 14 HH, and 14 HC subjects. We simple regression with hallucination level and median split analysis on this sample. Medication status did not differ between groups and was not correlated with any of the other neuropsychological or symptom barriers. Although the PANSS Hallucination Item does not distinguish between auditory hallucinations and hallucinations in other sensory modalities, 10 of the 15 subjects with scores of 4–6 reported that their hallucinations were “exclusively” auditory in nature, and 3 reported they were “mainly” auditory. For the response locked condition, applying the same quality controls to the response time windows winnowed the numbers to 26 SZ and 15 HC subjects due to additional movement artifacts during this task phase, leading to fewer than 60 usable trials in multiple SZ subjects.

For the purposes of visualizing the relationship between iHGP patterns and severity of hallucinations, schizophrenia subjects were divided based on a median split of their Hallucinations Item Score rating. We extracted the activation levels during stimulus encoding and pre-response periods for each subject in two pre-specified anatomically defined ROIs – posterior superior temporal gyrus (STGp) and visual word form area (VWFA). To reduce bias, the STGp location for each group was chosen as the voxel with maximum activation within a 10 mm radius from that found in our prior work, consistent with the localization of the functional area Spt^35,76^. Similarly, the VWFA location was chosen as the maximum voxel with a 10 mm radius of our main effect, consistent with the canonical location of VWFA^34^ and studies of individual variation in the VWFA location^46^. Functional activation within these independently defined anatomically ROIs were subject to one-way ANOVAs with groups: (HC), low-hallucination (LH), and high-hallucination (HH), as factors.

To assess neural-behavioral correlations, Pearson correlation coefficients were computed for activations/contrasts for all voxels against measures of accuracy. Statistical tests of performance were carried out on rationalized arcsine transformed (normalized) data^77^. P-values were corrected for multiple comparisons across voxels, frequency, and across time-points with the FDR procedure using a threshold of 5%.

## Data Availability

The data that support the findings of this study are available from the corresponding author upon reasonable request
